# Scientific Items

**Published:** 1879-02-20

**Authors:** 


### SCIENTIFIC ITEMS.

**New Inventions.**—An improved apparatus for use in photographic galleries, as an accessory in forming backgrounds for pictures, has been patented by Mr. William F. Ashe, of New York city. It is so constructed that it may be arranged as a staircase, a balustrade, or a pedestal, as may be required.

Messrs. Hiram D. Jewett and Israel D. Jewett, of St. Omer, Ind., have patented an improved mechanical telephone, which is so constructed as to receive the full volume and force of the voice and other sounds, magnify them, and transmit them to the instrument at the other end of the line with distinctness, so that they can be clearly heard at considerable distance from the instrument.

Mr. Wm. H. Hubbard, of Red Bank, N. J., has patented an improved gate, which consists of metal or wooden bars supported at one end in a post having a friction roller at the bottom running on a rail, and at the opposite end entered into metal or wooden tubes placed in or constituting the adjoining panel of the fence, so that when the gate is opened the rods of which it is made slide or telescope into the tubes of the adjoining panel.—*Scientific American.*

**The Perfection of Nature.**—Upon examining the edge of the sharpest razor with the microscope, it will appear fully as broad as the back of a knife—rough, uneven, and full of notches and furrows. An exceedingly small needle resembles an iron bar. But the sting of a bee seen through the same instrument exhibits everywhere the most beautiful polish, without a flaw, blemish, or inequality, and ends in a point too fine to be discerned. The threads of a fine lawn are coarser than the yarn with which ropes are made for anchors. But a silk worm's web appears smooth and shining, and everywhere equal. The smallest dot that is made with a pen appears irregular and uneven. But the little specks on the wings or bodies of insects are found to be an accurate circle. How magnificent are the works of Nature!—*Am. Inv.*

**Kidney Worm.**—Recent investigations indicate that the great kidney worm (*Strongylus gigas*) which exists in the kidneys of the horse, dog, and sometimes in man, lives in fishes in its young state; and the observations of Dr. Bertolus almost furnish proof that people contract the Swiss or broad tapeworm (*Bothriocephalus latus*) by feasting on improperly cooked trout. According to Professor Van Beneden, however, the latter parasite is at present known to occur only in Russia, Poland and Switzerland. All full-grown fishes sold in the shops as food are liable to contain entozoa.

**A Costly Telescope.**—M. Camille Flammarion, of Paris, has recently published a number of articles to prove that the moon is inhabited, and is now organizing a committee to collect the necessary funds to construct a refracting telescope of sufficient power to see them. He calculates the cost of the instrument at one million francs.—*Scientific American.*

**An Exhibition of Recent Inventions.**—An interesting exhibition of new apparatus was recently made at the rooms of the Royal Society, London. Among them were: The Mechanical Chameleon, to show the mixture of two colors in any proportion. A large Holtz electric machine (by Ladd), consisting of twelve rotary and twelve stationary plates, thirty inches diameter. A micro-spectroscope with improvements: (1) quick movement of the slide carrying the slit; (2) scale for registering position of slit; (3) arrangement for composing three spectra and for splitting a single spectrum; (4) new form of comparison stage made by Mr. A. Hilger. A dynamic-electric machine, speed 800 revolutions; power 1.75 horse-power required to work it; effect 1,200 candles' light, exhibited by Siemens Brothers. The telephone harp, with visible records of sounds through vacuum tubes. Apparatus for showing figures in light from vibrations caused by sound. A new metallic thermometer. An apparatus for the automatic registration of the number of hours of sunlight; and finally, the phonograph exhibited by Mr. Preece.—*Amer. Inventor.*

**Formation of Whirlwinds.**—In Herbert's recent communication to the French Academy, he has laid special stress upon the influence of the land configurations, and especially of the mountains and valleys, upon the great movements of the atmosphere. He comes to the conclusion that all the cyclones which visit Europe from the Atlantic Ocean originate in the American mountains. Many years ago Professor Henry indicated the region of the Saskatchawan as a prominent cradle of storms. More recent communications to the American Philosophical Society, based partly upon the observations of the Signal Service Bureau, and partly upon special meteorological records at San Francisco and at Barbadoes, have shown that there are two other important centres of extensive atmospheric disturbance—one in the neighborhood of Colorado, and another in the West India islands.—*Journal of Franklin Institute.*

**A Delicate Test for Arsenic.**—If any salt or compound of arsenic be put on a fibre of asbestos, and brought into the reducing point of a Bunsen burner, in which a little of the air has been cut off below, and a white china dish containing cold water held above the flame, the arsenious acid will be deposited as an invisible white film. This is moistened with a little neutral solution of silver nitrate, and then a trace of ammonia gas blown upon it from the stopper of an ammonia bottle. If any arsenic is present, a beautiful yellow color results, if antimony it is black, if both the black spot has a yellow border. On touching it with a drop of ammonia the yellow disappears, but the black remains. —*Boston Journal of Chemistry.*

**A New Blasting Agent.**—In Stockholm the following receipt has been given for a new blasting agent: In wooden or gutta-percha vessels 5 to 20 parts sugar or molasses are ground with 25 to 30 parts nitric acid, and 50 to 75 parts sulphuric acid. Of this mixture 25 to 50 parts are mixed with 15 to 35 parts nitrate of potassium and 15 to 35 parts cellulose. The agent is called nitrolin.—*Ib.*

